# Platelets are indispensable for alveolar development in neonatal mice

**DOI:** 10.3389/fped.2022.943054

**Published:** 2022-08-09

**Authors:** Zilu Huang, Bingchun Lin, Dongshan Han, Xuan Wang, Junyan Zhong, Gerry T. M. Wagenaar, Chuanzhong Yang, Xueyu Chen

**Affiliations:** ^1^Department of Neonatology, Affiliated Shenzhen Maternity and Child Healthcare Hospital, The First School of Clinical Medicine, Southern Medical University, Shenzhen, China; ^2^Faculty of Science, VU University Amsterdam, Amsterdam, Netherlands

**Keywords:** lung development, pulmonary hypertension, neonate, endothelial dysfunction, vascular remodeling

## Abstract

Previous studies suggest that platelets are involved in fetal and adult lung development, but their role in postnatal lung development especially after premature birth is elusive. There is an urgent need to scrutinize this topic because the incidence of bronchopulmonary dysplasia (BPD), a chronic lung disease after premature birth, remains high. We have previously shown impaired platelet biogenesis in infants and rats with BPD. In this study, we investigated the role of anti-CD41 antibody-induced platelet depletion during normal postnatal lung development and thrombopoietin (TPO)-induced platelet biogenesis in mice with experimental BPD. We demonstrate that platelet deficient mice develop a BPD-like phenotype, characterized by enlarged alveoli and vascular remodeling of the small pulmonary arteries, resulting in pulmonary arterial hypertension (PAH)-induced right ventricular hypertrophy (RVH). Vascular remodeling was potentially caused by endothelial dysfunction demonstrated by elevated von Willebrand factor (vWF) concentration in plasma and reduced vWF staining in lung tissue with platelet depletion. Furthermore, TPO-induced platelet biogenesis in mice with experimental BPD improved alveolar simplification and ameliorated vascular remodeling. These findings demonstrate that platelets are indispensable for normal postnatal lung development and attenuation of BPD, probably by maintaining endothelial function.

## Introduction

Lung development is a complex process that is stratified into five stages: embryonic, pseudoglandular, canalicular, saccular, and alveolar stage (1). Each stage is precisely regulated to ensure a functional lung at birth (2). Interfering with lung development during the early stages may lead to fetal death whereas interventions at later stages may compromise lung development and function later in life. These processes may be complicated by premature birth and treatment strategies are needed to improve aberrant lung development and function, thereby reducing morbidity and mortality (1). Bronchopulmonary dysplasia (BPD) is a chronic lung disease in extremely premature infants born in the canalicular to the saccular stage of lung development. These infants need mechanical ventilation and supplemental oxygen to survive (3). This may lead to a ventilator- and/or hyperoxia-induced trauma that complicates lung development and may lead to an arrest in alveolar development and comprised lung function (4).

Recently it was demonstrated that platelets are not only released by megakaryocytes in the bone marrow, but that the lung is also a major site for platelet production (5). New insights into platelet origin and production drove researchers to explore new thrombocyte functions (6). Tsukiji et al. found platelets are crucial for early lung development in the transition from the canalicular to saccular stage *via* regulation of lung mesothelial cell differentiation to myofibroblasts through interaction between Clec-2/podoplanin (7). Furthermore, platelets also contribute to neo-alveolarization after pneumonectomy in which stromal-cell-derived factor-1 receptors, CXCR4 and CXXR7 on pulmonary capillary endothelial cells are involved (8). However, there are fundamental differences in the complex processes that regulate early prenatal lung development, adult lung regeneration and postnatal lung development, including the developmental stage, injurious stimuli, contributing cell types and regulatory factors (1, 9). The role of platelets in lung development after the saccular stage and during the presence of external stimuli such as hyperoxia has not been elucidated yet.

Mice and premature infants at risk for BPD are born in the saccular stage of lung development, making mice appropriate models to study the transition from the saccular to the alveolar stage during normal development and BPD (10, 11). In our previous study, we found that platelet biogenesis increased after birth in both extremely premature infants and newborn rats, who share a similar lung developmental stage at birth. This increase in platelet biogenesis stabilizes at approximately day 20 of life in rats, which coincides with the process of postnatal alveolarization. These findings suggest a role for platelets in alveolarization in the developing lung (12). Furthermore, we also observed impaired platelet biogenesis in both BPD infants and rats with experimental BPD (12). In the current study, we investigated the role of platelets on lung development in newborn mice in which platelets are depleted using an anti-CD41 antibody during the saccular to alveolar transition period and in mice with experimental BPD in which reduced platelet production is stimulated by thrombopoietin (TPO).

## Materials and methods

### Animals

#### Platelet depletion models

**FIGURE 1:**
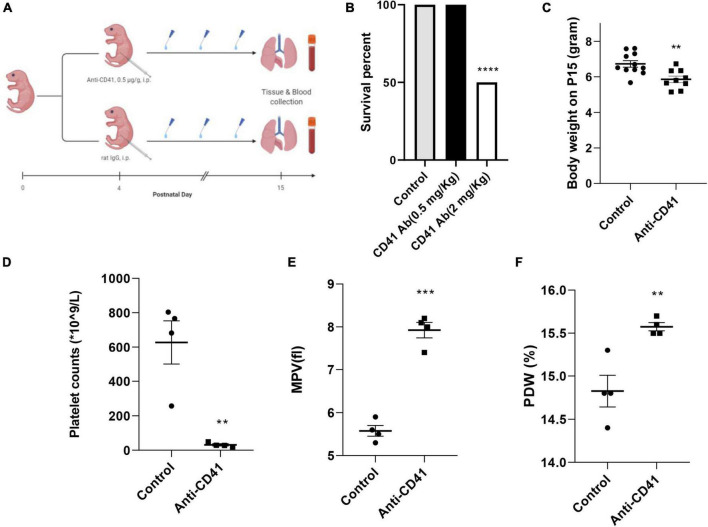
Effective platelet depletion with anti-CD41 antibody resulted in weight loss in newborn mice. Experimental scheme **(A)** of the newborn mice treated with anti-CD41 antibody or isotype (0.5 mg/Kg body weight, intraperitoneal) at postnatal day 4 (P4), P7, P10, and P13. Mice were sacrificed on day 15. Mortality **(B)** and body weight **(C)** on postnatal day 15 from mice in control (*n* = 11) and anti-CD41 group (*n* = 10). Platelet counts **(D)**, mean platelet volume **(E)** and platelet distribution width **(F)** in mice treated with anti-CD41 antibody or isotype, *n* = 4 in each group. Values are expressed as mean ± SEM, **p < 0.01, ***p < 0.001.

Newborn pups from five pregnant C57BL/6J mice (Jackson) were randomized into two groups: anti-CD41 vs. control (sacrificed on postnatal day 15) (*n* = 6 in each group). Pups were intraperitoneally injected either with a rat anti-mouse CD41 antibody (#553847, clone # MWReg30, BD Biosciences) or a rat IgG isotype control (#553922, clone # R3-34, BD Biosciences) at a dose of 0.5 mg/Kg every 3 days on days 4, 7, 10 and 13 to deplete the circulating platelets during the alveolarization period. The dose of anti-CD41 antibody is selected based on the best survival rate that can achieve a significant platelet depletion (Figure 1B). Anesthetized pups were sacrificed at the designated day after an intraperitoneal injection of pentobarbital (40 mg/Kg). Blood samples were drawn from the abdominal aorta, mixed with EDTA, and analyzed using a Mindray 5390 analyzer (Shenzhen, China) to acquire platelet parameters. Hereafter, lungs and hearts were harvested. Lung tissue was fixed *in situ* under the constant pressure of 26 cm H2O for 6 min with formalin or stored at −80°C until RNA extraction for RT-PCR. Three independent experiments were performed.

#### Thrombopoietin injection models

Newborn C57BL/6 mouse pups from 4 to 6 litters were pooled and assigned at random to 4 experimental groups: an oxygen-NaCl group (O2), an oxygen-TPO group (O2-TPO), and two room air (RA)-exposed control groups (*n* = 5–8 each). All oxygen-exposed pups were housed together in Plexiglas chambers and exposed to 95% oxygen from P3 to P11. Pups were fed by foster dams and received a daily subcutaneous injection with either 25 μg/Kg of recombinant murine TPO (# AF-315-14, Pepro Tech, Cranbury, NJ, United States) dissolved in 50 μl 0.9% NaCl or solvent from P4 to P10 (8). Foster dams were rotated daily (24 h in hyperoxia and 24 h in RA) to avoid oxygen toxicity. Anesthetized pups were sacrificed at P11 by intraperitoneal injection of pentobarbital (40 mg/kg). Blood was taken for platelet parameters analysis. Lungs were fixed in formalin for histology studies.

All animal experiments in this study were approved by the Institutional Animal Care and Use Committee of Shenzhen Institutes of Advanced Technology of the Chinese Academy of Sciences.

### Lung and heart morphometry

Lung and heart tissue was fixed in formalin and embedded in paraffin. Tissue sections (4 μm) were deparaffinized and subsequently stained with hematoxylin and eosin (HE), or primary antibodies against α-smooth muscle actin (α-SMA, #A2547, Sigma-Aldrich, St. Louis, MO, United States; diluted 1: 10,000) or von Willebrand factor (vWF, #A0082, Dako Cytomation, Glostrup, Denmark; diluted 1: 5,000). Tissue sections were further processed with HRP conjugated anti-mouse/rabbit secondary antibody (#ab6728 or #ab6721, Abcam, Cambridge, MA; diluted 1: 1,000), visualized using the chromogenic substrate NovaRed as recommended by the manufacturer (#SK-4800, Vector, Burlingame, CA, United States), and counterstained briefly with hematoxylin. Mean linear intercept (MLI) was used to assess alveolar development, as previously described (12). At least 1,000 alveoli per animal were measured. Quantification of secondary alveolar crests was determined on α-SMA-stained lung sections at a 400x magnification. The number of secondary crests in each section was counted in 10 non-overlapping fields and normalized to tissue area. Arteriolar medial wall thickness was assessed in α-SMA-stained lung sections at a 1,000x magnification. At least 10 vessels with a diameter of <30 μm were measured for each animal. Medial wall thickness was calculated from the formula “percent wall thickness = [(2 * wall thickness)/external diameter] * 100” (13). The number of arterioles per field was counted at a 200x magnification in vWF-stained lung sections to determine vascular density. At least 10 representative fields per animal were investigated. Fields with large blood vessels or bronchioles were excluded from the analysis. NIH Image J software was used for quantitative morphometry unless stated otherwise. Two independent researchers blinded to the experimental study groups performed the analysis.

Right and left free ventricular wall thickness, and interventricular septum thickness were assessed at a 40x magnification in HE-stained sections taken halfway the long axis. Six measurements per structure were made to acquire the average. Right ventricular hypertrophy (RVH) was calculated by dividing right ventricular (RV) free wall thickness with left ventricular (LV) free wall thickness. NIH Image J was used for analysis. Two independent researchers blinded to the exposure and treatment performed the analysis.

### Real-time PCR analysis

Total RNA was extracted from 30 mg lung tissue homogenates with TRIzol (#15596026, Invitrogen, Waltham, MA, United States) and 2 μg of total RNA was used for cDNA preparation by RevertAid First Strand cDNA Synthesis Kit (#K1622, Thermo Scientific, Waltham, MA, United States). Real-time PCR was performed in a 20-μl volume using an Applied Biosystems 7300Plus real-time PCR system (Applied Biosystems, Foster City, CA, United States). All RNA expression levels were normalized to 18S rRNA. Primers are listed in Supplementary Table 1.

### Enzyme-linked immunosorbent assay

Blood samples were centrifuged to remove blood cells and plasmas were collected. Next, von Willebrand factor (vWF) concentration was measured according to the manufacturer’s instructions of the vWF enzyme-linked immunosorbent assay (ELISA) kit (#E-EL-M1247c, Elabscience, Wuhan, China).

### Immunofluorescence staining

Deparaffinized lung tissue was subjected to either proteinase K solution (#ST533, Beyotime, Shanghai, China) or citrate buffer (pH 6.0) for antigen retrieval, then incubated with antibodies against vWF (#A0082, Dako Cytomation, Glostrup, Denmark; diluted 1: 2,000) or α-SMA (#A2547, Sigma-Aldrich, St. Louis, MO, United States; diluted 1: 5,000) overnight at 4°C. The tissue was further incubated with secondary antibodies Alexa Fluor Plus 555 anti-rabbit or anti-mice IgG fluorescent development (#A32732 and #A21422, Thermo Scientific, Waltham, MA, United States, diluted 1: 1,000), or TUNEL assay using the fluorescence-based TUNEL Kit (#C1088, Beyotime, Shanghai, China) according to the manufacturer’s instructions. Sections were mounted with a mounting solution with DAPI (#P0131, Beyotime, Shanghai, China), and visualized with a fluorescent microscope (IX73, Olympus, Tokyo, Japan) under an appropriative wavelength.

### Statistical analysis

Values are expressed as mean ± SEM unless stated otherwise and analyzed by student’s unpaired *t*-test. For statistical analysis GraphPad Prism version 8 software package was used (San Diego, CA, United States). A *p* < 0.05 was considered statistically significant.

## Results

### Anti-CD41 antibody treatment depleted circulating platelets and reduced body weight in newborn mice

Platelet depletion was studied in four neonatal mice per group after injections with the anti-CD41 antibody at a concentration of 0.5 mg/Kg for every 3 days on days 4, 7, 10, and 13 (Figure 1A). The dose was determined from a pilot experiment (Figure 1B). Pups subjected to platelet depletion showed a minor but significant reduction in body weight (5.8 vs. 6.7 g, *p* < 0.01, Figure 1C). On day 15, platelet counts in blood samples were significantly decreased by 20-fold (31 vs. 627 × 10^9^/L, *p* < 0.01, Figure 1D), but mean platelet volume (MPV) and platelet distribution width (PDW) were significantly increased (7.93 vs. 5.58 fl, *p* < 0.001, Figure 1E and 15.58 vs. 14.83%, *p* < 0.01, Figure 1F, respectively) after repeated anti-CD41 antibody injections compared with controls.

### Platelet depletion impaired lung alveolarization and caused vascular remodeling and right ventricular hypertrophy, but did not affect angiogenesis in neonatal mice

**FIGURE 2:**
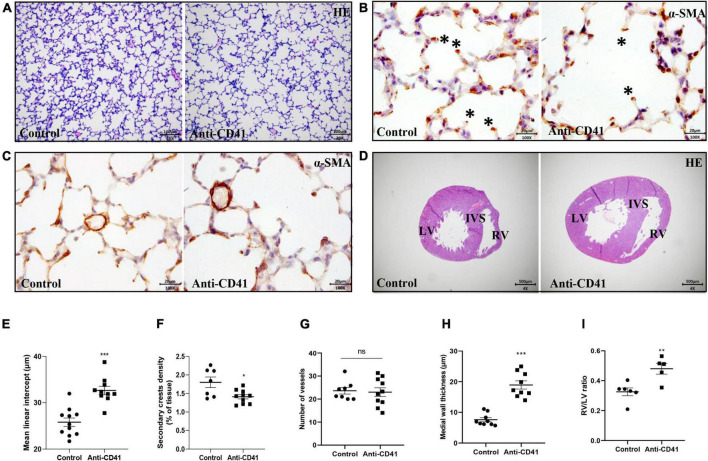
Platelet depletion impaired lung alveolarization and caused vascular remodeling but did not affect angiogenesis in neonatal mice. Representative images of hematoxylin-eosin (HE, **A**) and α-smooth muscle actin (α-SMA, **B,C**) stained lungs from pups treated with anti-CD41 antibody or isotype. Representative images of HE-stained hearts taken halfway the long axis **(D)**. Quantification of mean linear intercept (MLI, **E**), secondary crest density corrected for tissue area **(F)**, number of vessels **(G)**, medial wall thickness **(H)** and right ventricular hypertrophy (RVH, **I**). Data are expressed as mean ± SEM, **p < 0.01. ***p < 0.001. Asterisks indicate secondary crests.

To ascertain the critical role of platelets in postnatal lung alveolar and vascular development, we evaluated MLI and secondary alveolar crests density in control pups and in anti-CD41 antibody platelet depleted pups during postnatal development. Platelet depleted pups showed alveolar enlargement, demonstrated by increased MLI (32.66 vs. 25.81 μm, *p* < 0.001, Figures 2A,E) and reduced secondary crests density (1.3-fold, *p* < 0.05, Figures 2B,F) compared with controls, but did not reduce the number of arterioles in vWF stained lung sections between the two groups (Figure 2G and Supplementary Figure 2C).

Furthermore, platelet depletion resulted in vascular remodeling demonstrated by increased pulmonary arterial medial wall thickness (2.5-fold, *p* < 0.001), determined on α-SMA-stained sections (Figures 2C,H) and caused right ventricular hypertrophy (RVH), demonstrated by a significantly increase in the ratio RV/LV free wall thickness (1.5-fold, *p* < 0.01, Figures 2D,I).

### Platelet depletion results in endothelial dysfunction in neonatal mice

**FIGURE 3:**
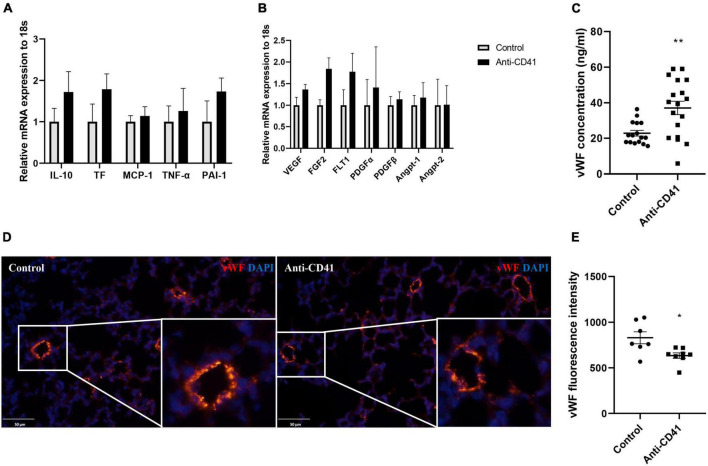
Platelet depletion disrupted endothelial function. mRNA in whole lung tissues was obtained from mice injected with anti-CD41 and control and analyzed by RT-PCR. Expressions of interlukin-10 (IL-10), tissue factor (TF), monocyte chemoattractant protein-1 (MCP-1), tumor necrosis factor –α (TNF-α), plasminogen activator inhibitor –1 (PAI-1) were relatively quantified in **(A)**, expressions of vascular endothelial growth factor (VEGF), fibroblast growth factor 2 (FGF2), VEGF receptor 1 (FLT-1), platelet-derived growth factor α (PDGFα), PDGFβ, angiopoietin 1 (Angpt-1), and Angpt-2 were relatively quantified in **(B)**, using 18S rRNA as a reference control (*n* = 6–8). Quantification of plasma concentration of vWF **(C)** in controls (*n* = 16) and mice injected with anti-CD41 (*n* = 18). Representative images of vWF staining in lung tissue **(D)** and quantification of vWF fluorescence intensity **(E)** in controls (*n* = 7) and mice injected with anti-CD41 (*n* = 8). Data are expressed as mean ± SEM, *p < 0.05, **p < 0.01.

We next investigated the potential mechanisms of platelet depletion on aberrant lung development and vascular remodeling. Although mRNA expression of inflammatory and endothelial markers was not significantly different, expression of all markers showed a tendency toward higher levels after platelet depletion compared with controls (Figures 3A,B). In addition, we observed increased levels of plasma vWF in platelet depleted mice (37 vs. 23 ng/ml, *p* < 0.01; Figure 3C), a factor synthesized by and stored in endothelial cells and released into the plasma when the vascular endothelium is damaged, indicating endothelial dysfunction in these pups. In line with this finding, we confirmed decreased vWF intensity in the mice lung with platelet depletion (*p* < 0.05; Figures 3D,E). Endothelial dysfunction was further supported by pulmonary hemorrhage observed in mice with platelet depletion (Supplementary Figure 1A).

### Thrombopoietin-induced platelet production attenuates experimental bronchopulmonary dysplasia

**FIGURE 4:**
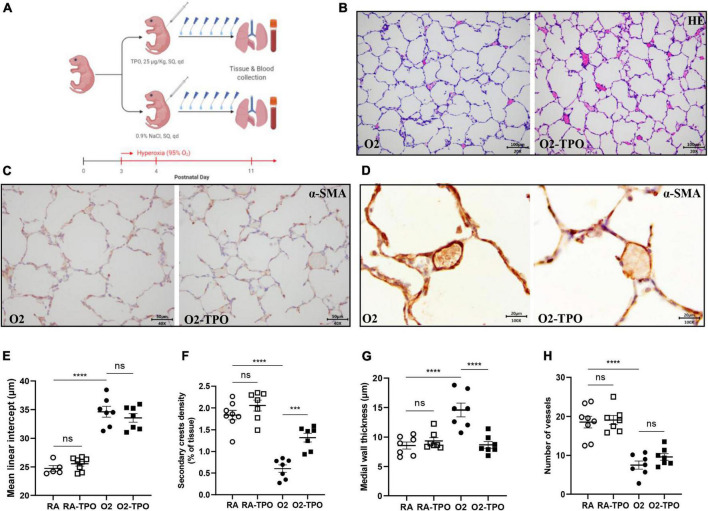
TPO attenuates experimental BPD. Experimental scheme **(A)** of the newborn mice exposed to hyperoxia and treated with TPO or NaCl once a day from postnatal day 4 to 10. Representative images of HE **(B)** and α-SMA **(C,D)** stained lungs. Quantification of MLI determined on HE stained lung **(E)**, secondary crest density corrected by tissue area determined on α-SMA stained lung **(F)**, medial wall thickness **(G)** and the number of vessels **(H)**. Data are expressed as mean ± SEM, ***p < 0.001, ****p < 0.0001.

We have previously shown that platelets are reduced in infants and rats with BPD (12). In this study, we demonstrated that platelet depletion in mice led to alveolar simplification with enlarged alveoli, similar to experimental BPD (Figures 2E,F). These data prompted us to investigate whether elevating the number of platelets by TPO injection would preserve lung development in mice with hyperoxia induced BPD. The treatment scheme is shown in Figure 4A.

Injection of TPO significantly increased the number of circulating platelets compared with hyperoxia-exposed controls (1814 vs. 820 × 109/L, *p* < 0.001, Supplementary Figure 1C). This increase in platelets was associated with a tendency towards a reduction in MLI (Figures 4B,E) and an increased number of secondary crests (2.2-fold, *p* < 0.001; Figures 4C,F). Similarly, TPO treatment significantly attenuated vascular remodeling caused by hyperoxia, as demonstrated by reduced medial wall thickness (1.4-fold, *p* < 0.001; Figures 4D,G), but pulmonary vessel density did not change significantly (*p* > 0.05; Figure 4H).

## Discussion

In the current study, we investigated the role of platelets during early postnatal lung development and experimental BPD by targeting platelet loss and gain in mice. In platelet deficient mouse pups, we observed a BPD phenotype characterized by enlarged or simplified alveoli and a reduced number of secondary crests, accompanied by small arterial remodeling and RVH, that may be caused by endothelial dysfunction after platelet depletion. Furthermore, we also studied the effect of TPO-induced “platelet gain” as a treatment option for BPD in pups with reduced platelets caused by exposure to hyperoxia. TPO significantly increased the number of secondary crests and reduced vascular remodeling in mice with experimental BPD, indicating that platelets are indispensable for postnatal lung development.

The mechanisms by which platelets interfere with postnatal lung development remains to be elucidated. Tsukiji et al. have previously demonstrated that platelet activation *via* C-type lectin-like receptor-2 (CLEC-2) regulated differentiation of alveolar duct myofibroblasts (adMYF) from lung mesothelial cells (luMCs) (7), which is essential for mouse fetal lung development. Noticeably, they focused on much earlier stages of fetal lung development ranging from the pseudoglandular to the early saccular stage from E10.5 to birth, while we focused on postnatal lung development from the saccular to the alveolar stage in our study. Despite this difference in the phase of lung development between our study and that of Tsukiji et al., aberrant myofibroblast differentiation may affect lung development in platelet depleted pups after birth. In line with this concept, we observed a decreased number of secondary crests, aligning with number of myofibroblasts (visualized by α-SMA staining) in platelet-depleted mice, which is further supported by a significant decrease in pulmonary α-SMA fluorescence intensity in platelet-depleted mice (Supplementary Figures 2A,B). Considering the crucial role of myofibroblasts in the development of secondary crests (14), the role of platelets on myofibroblasts in postnatal lung development warrants further investigation.

Furthermore, Rafii et al. suggested that platelets are involved in lung regeneration by stimulating pulmonary capillary endothelial cells (8). Surprisingly, neither loss of platelets nor gain of platelets during postnatal development significantly affected pulmonary vascular density in the current study. This discrepancy in vascular development could be explained by the fact that vWF is missing in capillary endothelial cells, which is the key mediator in platelet-driven lung regeneration (8). Although another endothelial marker CD31, which is expressed on capillaries, could be a better option, the quantification of capillaries is rather difficult in practice. Therefore, the gap in our knowledge in the involvement of vascularization in platelet-associated postnatal lung development has to be bridged in further studies. Furthermore, there are fundamental differences between lung development and lung regeneration. For example, alveolar epithelial type I cells (AECI) and alveolar epithelial type II cells (AECII) are derived from a bipotent progenitor in postnatal lung development, while AECII are a major source of AECI for tissue repair in the adult lung (15–17).

Although a direct causality between increased pulmonary platelets and the beneficial effect of TPO in hyperoxia exposed mice was not established in this study, we assume that the increased platelet numbers in the systemic circulation will contribute to increased platelet numbers in the lung vascular bed as well. In addition, it has been shown that TPO, the key mediator of megakaryocyte maturation, increased numbers of megakaryocytes and proplatelets in the pulmonary vascular bed (5, 18). Therefore, we infer the beneficial effect of TPO in experimental BPD was mediated by an increase of platelets in the lung. Both Rafii et al. and Tsukiji et al. indicated that activation of platelets is essential for normal lung development and beneficial in experimental BPD (7, 8). We also observed an increased mean platelet volume after platelet depletion, suggesting that platelet activation and platelet numbers may be important players in proper lung development and disease. Therefore, whether the activation or the number of platelets is the key mediators warrants further investigation.

We are the first to show that postnatal platelet depletion leads to pulmonary vascular remodeling and RVH, which is probably caused by vascular endothelial dysfunction, which has long been linked to PAH (19). A pivotal role of platelets in maintaining endothelial function is further suggested by our experimental data showing that platelet depletion resulted in lung hemorrhage, thereby confirming previous data (20). The PAH and RVH observed in platelet depleted mice prompted us to reflect the causality between PAH and BPD. In both premature infants and experimental rodent BPD models, the onset or diagnosis of PAH is later than that of BPD; so-called BPD-associated PAH (21). Evidence is increasing that the onset of PAH in preterm infants occurs within 1 week after birth (22, 23), thereby raising the hypothesis that early pulmonary vascular disease could prime the development of BPD (23). The observed aberrant lung development after platelet depletion in the current study could be attributed to pulmonary vascular dysfunction, demonstrated by increased vascular remodeling which resulted in RVH.

We found that administration of TPO improved aberrant alveolar development in mice with hyperoxia-induced BPD by increasing secondary crests density. However, MLI was not significantly improved, but only showed a tendency to decreased levels. Improvements in the number of secondary crests precede MLI. Therefore, we expect an improvement in MLI as well after a longer period of TPO treatment. Although our data are very promising, further studies are needed to confirm the therapeutic potential of TPO in premature infants with BPD.

We acknowledge several limitations in this study. First, the causality between PAH and arrested alveolarization was not established, which might be of clinical relevance in managing infants with a high risk of developing BPD. Furthermore, although it is common to expose mouse pups to hyperoxia soon after birth to establish experimental BPD, we had to delay the exposure to hyperoxia to P3 due to high mortality rates in the first 2 days after birth. However, this late onset of exposure to hyperoxia is appropriate to induce lung morphometric changes resembling BPD pathology in the transition period from the saccular to the alveolar stage of lung development (24). Besides, there is a minor but significant decrease in body weight in pups with platelet depletion, suggesting a potential interference of poor nutrition on arrested lung development, which cannot be excluded in this study.

Taken together, we have demonstrated an indispensable role of platelets in the developing lung and the therapeutic potential of platelet stimulation by TPO in preventing or attenuating BPD. Further studies are needed to confirm these findings.

## Data availability statement

The original contributions presented in this study are included in the article/Supplementary material, further inquiries can be directed to the corresponding authors.

## Ethics statement

The animal study was reviewed and approved by the Institutional Animal Care and Use Committee of Shenzhen Institutes of Advanced Technology of the Chinese Academy of Sciences.

## Author contributions

CY and XC conceptualized and designed the study. ZH wrote the first draft of the manuscript. DH and XW carried out the experiments. ZH, BL, and JZ performed the data analysis. GW, CY, and XC reviewed and revised the manuscript. All authors read and approved the final manuscript.

## Supplementary material

Supplementary Figure 1 Representative images of pulmonary hemorrhage in mice receiving anti-CD41 antibody **(A)**. Representative images of apoptotic endothelium visualized by TUNEL and vWF col-localization **(B)**. Platelets count measured in 0.9% NaCl or TPO treated mice pups exposed to hyperoxia **(C)**. Representative images of HE **(D)** and α-SMA **(E,F)** stained lungs from mice raised in room air.

Supplementary Figure 2 Representative images and quantification of α-SMA fluorescence staining **(A,B)** in lung tissue from controls (*n* = 5) and mice injected with anti-CD41 antibody (*n* = 5). Representative images of vWF staining **(C)**. Data are expressed as mean ± SEM, ****p* < 0.001.
