# Supplementary material for: Platelets are indispensable for alveolar development in neonatal mice
Source: Front Pediatr. 2022 Aug 9;10:943054. doi: 10.3389/fped.2022.943054 (PMC9396244; doi:10.3389/fped.2022.943054)
Supplement: Supplementary file 1 [file Table_1.DOCX]

Supplemental Table 1 Sequences of oligonucleotides of forward and reverse primers for RT-PCR

| Gene | Forward primer | Reverse primer |
| --- | --- | --- |
| IL-10 | 5’-CCCATTCCTCGTCACGATCTC-3’ | 5’-TCAGACTGGTTTGGGATAGGTTT-3’ |
| TF | 5’-CAATGAATTCTCGATTGATGTGG-3’ | 5’-GGAGGATGATAAAGATGGTGGC-3’ |
| MCP-1 | 5’-GCTCTCTCTTCCTCCACCACCAT-3’ | 5’-GCTCTCCAGCCTACTCATTGGGAT-3’ |
| TNF-α | 5’-AAGCCTGTAGCCCACGTCGTA-3’ | 5’-GGCACCACTAGTTGGTTGTCTTTG-3’ |
| PAI-1 | 5’-ACGCCTGGTGCTGGTGAATGC-3’ | 5’-ACGGTGCTGCCATCAGACTTGTG-3’ |
| VEGF | 5'-AACGAAAGCGCAAGAAATCC-3' | 5'-GCTCACAGTGAACGCTCCAG-3' |
| FGF2 | 5’-GAACCGGTACCTGGCTATGA-3’ | 5’-CCGTTTTGGATCCGAGTTTA-3’ |
| FLT1 | 5’-AATAAGACGGTTAGCACATTGGT-3’ | 5’-TCCTCTCCTTCGGTTGGTATC-3’ |
| PDGFα | 5'-AAGCATGTGCCGGAGAAGCG-3' | 5'-TCCTCTAACCTCACCTGGAC-3' |
| PDGFβ | 5'GAAGCCAGTCTTCAAGAAGGCCAC-3' | 5'AACGGTCACCCGAGTTTGAGGTGT-3' |
| Angpt-1 | 5’-GGGGGAGGTTGGACAGTAA-3’ | 5’-CATCAGCTCAATCCTCAGC-3’ |
| Angpt-2 | 5’-GATCTTCCTCCAGCCCCTAC-3’ | 5’-TTTGTGCTGCTGTCTGGTTC-3’ |
| 18S rRNA | 5’-CGGCTACCACATCCAAGGAA-3’ | 5’-GCTGGAATTACCGCGGCT-3’ |
