# Supplementary figures and images for: Platelets are indispensable for alveolar development in neonatal mice
Source: Front Pediatr. 2022 Aug 9;10:943054. doi: 10.3389/fped.2022.943054 (PMC9396244; doi:10.3389/fped.2022.943054)

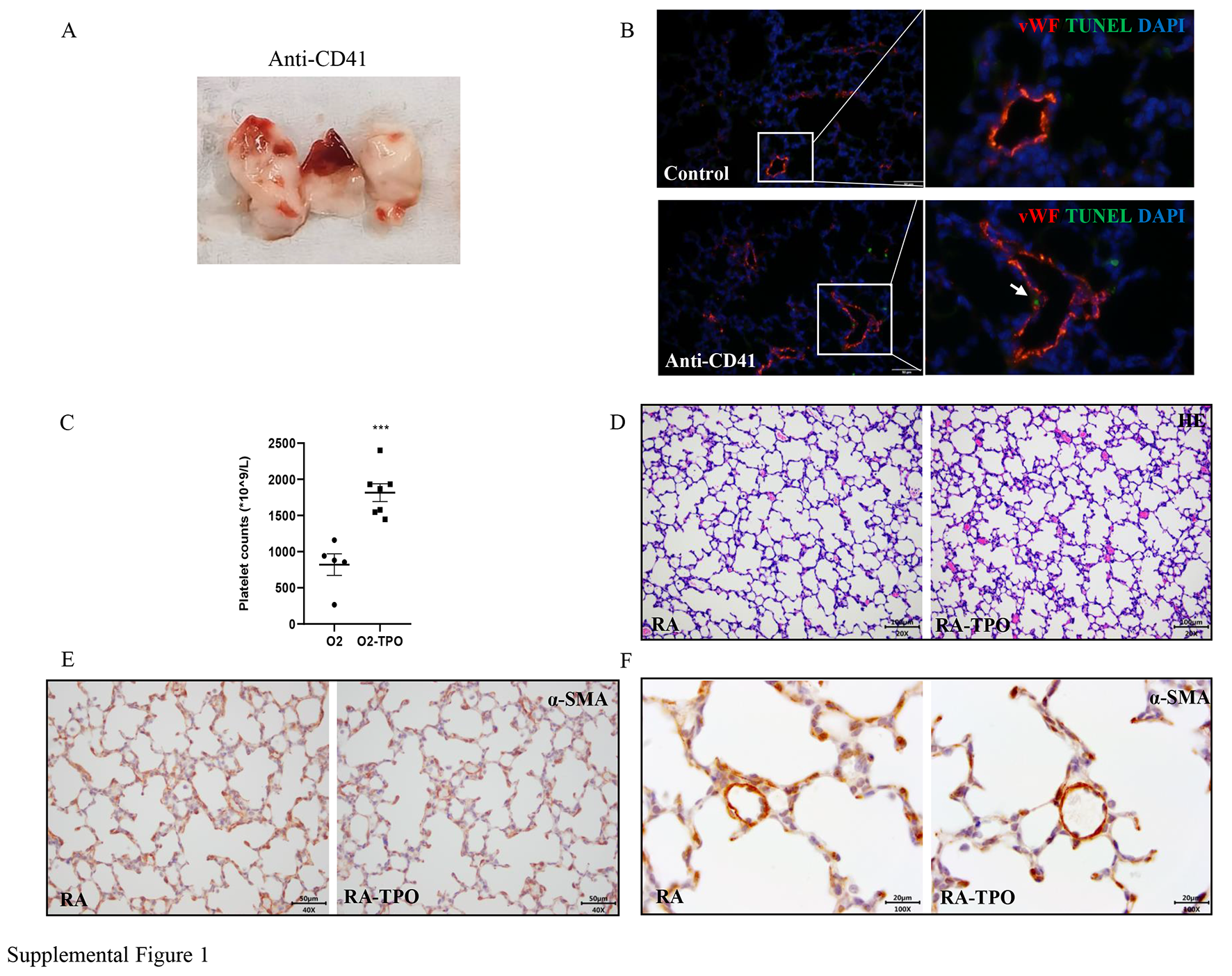

Supplement: Supplementary Figure 1 — Representative images of pulmonary hemorrhage in mice receiving anti-CD41 antibody (A). Representative images of apoptotic endothelium visualized by TUNEL and vWF col-localization (B). Platelets count measured in 0.9% NaCl or TPO treated mice pups exposed to hyperoxia (C). Representative images of HE (D) and α-SMA (E,F) stained lungs from mice raised in room air. [file Image_1.TIF]

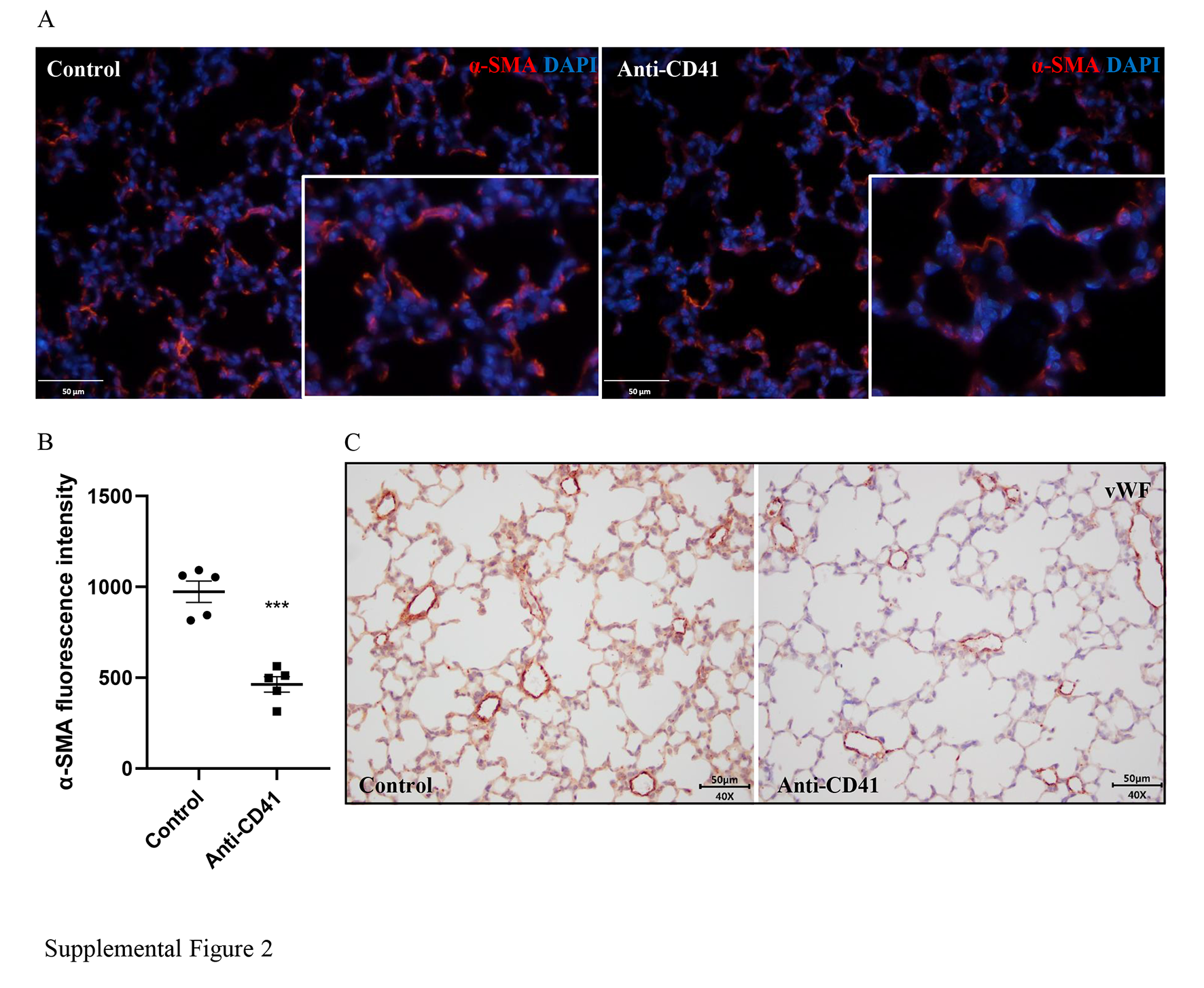

Supplement: Supplementary Figure 2 — Representative images and quantification of α-SMA fluorescence staining (A,B) in lung tissue from controls (n = 5) and mice injected with anti-CD41 antibody (n = 5). Representative images of vWF staining (C). Data are expressed as mean ± SEM, ***p < 0.001. [file Image_2.TIF]
